# Development and evaluation of clinical pregnancy prediction models for intrauterine insemination using three machine learning algorithms

**DOI:** 10.3389/fendo.2026.1749988

**Published:** 2026-04-24

**Authors:** Yingwei Fu, Dazhi Li, Xi Xia, Changzhong Li, Junjie Wang, Chunhui Zhang

**Affiliations:** 1 Department of Gynecology, The People’s Hospital of Baoan Shenzhen, Shenzhen, China; 2 Department of Reproductive Medicine, Peking University Shenzhen Hospital, Shenzhen, Guangdong, China; 3 Department of Gynecology, Peking University Shenzhen Hospital, Shenzhen, Guangdong, China

**Keywords:** clinical pregnancy, infertility, intrauterine insemination, machine learning, multilayer perceptron, prediction model

## Abstract

**Background:**

Predicting clinical pregnancy following intrauterine insemination (IUI) remains challenging because of the complex interplay of multiple biological and treatment-related factors. Machine learning approaches may improve predictive accuracy compared with conventional statistical methods. This study aimed to compare the predictive performance of logistic regression (LR), random forest (RF), and multilayer perceptron (MLP) models and to explore their clinical interpretability.

**Methods:**

A total of 957 IUI cycles were randomly divided into a training set (80%) and an independent validation set (20%). Synthetic Minority Oversampling Technique (SMOTE) was applied to the training set to address class imbalance. Model performance was evaluated using 10-fold cross-validation, and discrimination was assessed by area under the receiver operating characteristic curve (AUC). Model interpretability was examined using SHAP (Shapley Additive Explanations) analysis.

**Results:**

Across 10-fold cross-validation, all three models demonstrated modest and comparable discriminative ability. The MLP achieved a mean AUC of 0.547 (cross-validation mean AUC 0.560 ± 0.062), while RF and LR yielded mean AUC values of 0.549 and 0.541, respectively. Although overall discrimination was limited, the MLP showed relatively greater stability across folds. SHAP analysis consistently identified female age, infertility etiology, body weight, antral follicle count, estradiol, and treatment regimen as important predictors across models.

**Conclusion:**

The MLP model showed modest discriminative ability in predicting IUI clinical pregnancy and identified several relevant clinical features. While its predictive performance is limited, the model may contribute to risk stratification and exploratory individualized assessment in assisted reproduction.

## 1 Introduction

Intrauterine insemination (IUI) is widely accepted by infertile couples because of its simplicity, safety, affordability, and relatively short treatment cycle. However, its clinical pregnancy rate remains modest. An accurate prediction of IUI success before treatment could help shorten the time to conception, reduce patients’ emotional burden, and support clinicians in making personalized treatment decisions.

In recent years, the application of artificial intelligence (AI) in medicine has expanded rapidly, including in reproductive medicine. AI-based approaches have been applied to sperm identification and selection, gamete screening, embryo development monitoring using multimodal imaging, embryo quality assessment, and studies of molecular mechanisms underlying embryo development (1, 2). Moreover, AI models have been developed to predict live birth outcomes after frozen–thawed embryo transfer (3), based on blastocyst morphology and developmental parameters (4, 5). Most previous prediction studies have relied on logistic regression or random forest algorithms, whereas neural network models have been less frequently explored. Neural networks, inspired by the human brain’s information processing mechanisms, consist of interconnected computational units (neurons) organized into input, hidden, and output layers. Through iterative learning, these models can capture complex nonlinear relationships within clinical data and imaging information (6).

The prediction of IUI outcomes has long relied on traditional clinical models, including logistic regression (LR) and random forest (RF), which use factors such as age, follicle count, and hormone levels. However, these models often fail to capture the non-linear interactions between variables, which are critical for predicting IUI success. Recent studies have started to explore the application of machine learning models, such as support vector machines and artificial neural networks, in this context. Yet, the use of multilayer perceptions (MLP), a type of neural network, has been largely underexplored in IUI prediction. This study aims to bridge this gap by comparing MLP, RF, and LR models on a dataset of IUI cycles, providing insights into how MLP can improve predictive performance over traditional models.

## 2 Materials and methods

### 2.1 Subjects

This retrospective study analyzed 957 IUI cycles performed at the Reproductive Medicine Center of Peking University Shenzhen Hospital between January 2022 and October 2024. Eligible participants met the following criteria: unilateral or bilateral tubal patency confirmed by hysterosalpingo-contrast sonography, hysterosalpingography, or hystero-laparoscopy; a total motile sperm count greater than 10×10^6^/mL; one or two dominant follicles with a diameter of at least 14 mm on the day of human chorionic gonadotropin (hCG) administration; and no more than three IUI treatment attempts. Women with systemic or genetic disorders, premature ovarian failure, recurrent miscarriage, severe endometriosis, or those receiving donor insemination were excluded.

### 2.2 Treatment protocol

According to ovulation status, treatment cycles were classified as natural or ovarian stimulation (OS) cycles. Ovulation induction regimens included clomiphene citrate (CC), letrozole (LE), CC or LE combined with gonadotropins, or gonadotropins alone, such as follicle-stimulating hormone (FSH) and human menopausal gonadotropin (HMG). Women with regular ovulatory cycles were monitored naturally, while those with ovulatory dysfunction received 5 mg of LE or 100 mg of CC orally for five consecutive days beginning on days 2–3 of the menstrual cycle. Follicular growth was monitored by transvaginal ultrasound, followed by injections of 75 IU FSH or HMG every other day or daily as required. When the leading follicle reached a diameter greater than 18 mm, 6000 IU of hCG was administered to trigger ovulation. Cycles with three or more follicles of 16 mm or greater were canceled to prevent multiple pregnancies.

Insemination was performed 12–36 hours after hCG injection. Following the procedure, patients were instructed to rest in a supine position for 20 minutes. Transvaginal ultrasound was repeated the next day to confirm ovulation. If ovulation was confirmed, 100 mg of oral progesterone was administered twice daily for 14 days to support the luteal phase, and treatment was continued to the 10th week of gestation in cases of successful conception. Semen samples were collected by masturbation after three days of abstinence and processed according to the World Health Organization’s fifth edition laboratory manual. After liquefaction, samples were optimized by density gradient centrifugation combined with the swim-up technique to obtain a 0.3–0.5 mL suspension of motile sperm for insemination. Clinical pregnancy was defined as visualization of an intrauterine gestational sac with fetal heartbeat by transvaginal ultrasound at eight weeks of gestation.

### 2.3 Statistical methods

A total of 28 clinical and laboratory variables were collected, including demographic, endocrine, and reproductive parameters such as female and male age, infertility duration and type, body mass index (BMI), menstrual characteristics, basal hormone levels (AMH, FSH, LH, E2, P, T, and PRL), antral follicle count, number and size of follicles on the day of hCG administration, endometrial thickness, post-treatment sperm concentration, and treatment regimen. The outcome variable was defined as a binary indicator of clinical pregnancy (pregnant = 1, not pregnant = 0). Variables with missing rates exceeding 15% were excluded. For the remaining data, missing values in continuous variables were imputed using mean substitution, while categorical variables were imputed using the mode.

The dataset comprising 957 IUI cycles was randomly divided into a training set (80%, n = 764) and an independent test set (20%, n = 193). To address class imbalance in the training data, the Synthetic Minority Oversampling Technique (SMOTE) was applied exclusively to the training set, generating synthetic minority-class samples to balance the distribution of pregnancy and non-pregnancy outcomes. Importantly, no oversampling was performed on the test set to avoid data leakage and optimistic bias. After SMOTE processing, the resampled training set included 1,175 cases and was used solely for model development, whereas the test set remained unchanged for model evaluation.

Three predictive models were constructed using logistic regression (LR), random forest (RF), and multilayer perceptron (MLP) algorithms. The RF model was built using 100 trees with a maximum tree depth of 10, and model growth was stopped when no further improvement in accuracy was observed. The MLP model was trained using stratified 10-fold cross-validation, allowing assessment of model stability across folds and operating thresholds. Final model predictions were summarized across folds to reduce dependence on a single data split.

Model performance was evaluated on the independent test set using accuracy, balanced accuracy, sensitivity, specificity, and the area under the receiver operating characteristic curve (AUC). Rather than relying on formal hypothesis testing for pairwise model comparison, performance variability and consistency across cross-validation folds and decision thresholds were examined to assess relative model behavior. SHAP analysis was performed to evaluate feature contributions for MLP and RF models using a unified model-agnostic framework.

All statistical analyses were conducted using IBM SPSS Statistics (version 27.0) and IBM SPSS Modeler (version 18.0). Receiver operating characteristic (ROC) curve analyses were performed in R using the pROC package (version 1.18.0). For SHAP computation, the TreeExplainer algorithm was applied to the random forest model, while the KernelExplainer was used for the multilayer perceptron model.

## 3 Results

After data processing, a total of 957 IUI cycles were included in the final analysis, with an overall clinical pregnancy rate of 16.8%. The mean age of female and male participants was approximately 32 and 34 years, respectively. The average duration of infertility was about three years. Most women had regular menstrual cycles and normal baseline hormone levels. Secondary infertility was more common than primary infertility, accounting for nearly two-thirds of all cases. Regarding etiological classification, female factors were identified in about one-third of cases, male factors in one-fourth, and unexplained infertility in approximately 40%. Detailed baseline characteristics of the study population are summarized in **Table 1**.

**Table 1:** Baseline characteristics of patients undergoing IUI (N = 957).

Continuous variables	Mean± SD	Categorical variables	n (%)
Female age (years)	32.16 ± 3.52	Type of infertility	
Male age (years)	34.05 ± 4.07	Primary infertility	668 (69.8)
Female height (m)	1.60 ± 0.05	Secondary infertility	289 (30.2)
Female weight (kg)	54.80 ± 7.86	Etiology of infertility	
Age at menarche (years)	12.57 ± 0.51	Female factor	279 (29.2)
Menstrual duration (days)	6.02 ± 1.54	Male factor	236 (24.7)
Menstrual cycle (days)	37.64 ± 23.07	Both factors	41 (4.3)
FSH (IU/L)	7.56 ± 2.54	Unexplained infertility	401 (41.9)
LH (IU/L)	5.54 ± 3.59	Tubal patency	
Estradiol (E2, pg/ml)	33.98 ± 12.32	Bilateral patent	856 (89.4)
Progesterone (P, ng/mL)	0.51 ± 0.33	Bilateral obstruction	8 (0.8)
Testosterone (T, ng/mL)	0.62 ± 0.68	Right patent, left obstructed	31 (3.2)
Prolactin (PRL, ng/mL)	16.14 ± 8.16	Right patent, left hydrosalpinx	24 (2.5)
Antral follicle count (AFC-R)	8.50 ± 2.98	Left patent, right obstructed	24 (2.5)
Antral follicle count (AFC-L)	8.27 ± 3.05	Left hydrosalpinx, right patent	13 (1.4)
Post-wash PR-grade sperm concentration (×10^6^/mL)	24.05 ± 18.33	Left and right hydrosalpinx	1 (0.1)
Dominant follicle size (mm)	19.60 ± 2.51	Treatment regimen	
Endometrial thickness on hCG day (mm)	10.33 ± 2.26	Natural cycle	300 (31.4)
Duration of infertility (years)	2.88 ± 1.96	Ovarian stimulation cycle	657 (68.6)
BMI (kg/m²)	21.29 ± 2.96	Post-wash sperm density	
AMH (ng/mL)	4.80 ± 3.29	Below standard	902 (94.3)
		Standard	55 (5.7)
		No. of follicles ≥14 mm on HCG day	
		1	745 (77.9)
		2	212 (22.1)
		No. of IUI procedures	
		1	409 (42.7)
		2	548 (57.3)

As shown in **Table 2**, the MLP model yielded the highest number of correctly predicted pregnancies (14/33, 42.4%) while also maintaining strong classification of non-pregnant cases (127/160, 79.4%). RF and LR showed comparatively lower true-positive rates for clinical pregnancy (36.4% and 33.3%, respectively).

**Table 2:** Confusion matrices and corresponding classification metrics of MLP, RF, and LR models in the IUI test set.

Algorithm	Actual outcome	Predicted non-pregnant	Predicted clinical pregnancy
MLP	Non-pregnant (n=160)	127 (79.4)	33 (20.6)
	Clinical pregnancy (n=33)	19 (57.6)	14 (42.4)
	Total (n=193)	146 (75.6)	47 (24.4)
RF	Non-pregnant (n=160)	121 (75.6)	39 (24.4)
	Clinical pregnancy (n=33)	21 (63.6)	12 (36.4)
	Total (n=193)	142 (73.6)	51 (26.4)
LR	Non-pregnant (n=160)	119 (74.4)	41 (25.6)
	Clinical pregnancy (n=33)	22 (66.7)	11 (33.3)
	Total (n=193)	141 (73.1)	52 (26.9)

MLP, Multilayer perceptron; RF, Random forest; LR, Logistic regression.

**Table 3** summarizes the predictive performance of the models. On the independent test set, the MLP showed slightly higher balanced accuracy than RF and LR, while overall performance remained modest, with an accuracy of 73.06%, balanced accuracy of 60.09%, sensitivity of 42.42%, and specificity of 79.38%. RF showed moderate performance, while LR had the lowest accuracy and balanced accuracy. Overall, the MLP provided the most favorable balance between sensitivity and specificity. In addition, the results of 3 models without SMOTE-based class balancing were showed as **Supplementary Tables 1** and **2**.

**Table 3:** Accuracy, balanced accuracy, sensitivity, and specificity of the MLP, RF, and LR models in the IUI test set.

Algorithm	Accuracy (%)	Balanced accuracy (%)	Sensitivity (%)	Specificity (%)
MLP	73.06	60.09	42.42	79.38
RF	68.91	55.99	36.36	75.63
LR	67.36	53.85	33.33	74.38

MLP, Multilayer perceptron; RF, Random forest; LR, Logistic regression.

The performance of the MLP, RF, and LR models was evaluated using 10-fold cross-validation. For each model, the mean and standard deviation of AUC, sensitivity, specificity, and balanced accuracy across folds were calculated. The MLP model consistently demonstrated slightly higher performance than RF and LR, particularly in AUC and balanced accuracy. The differences in AUC across models were modest, with largely overlapping ranges across folds. The performance ranges across folds largely overlapped, indicating modest differences between models.

**Figure 1:**
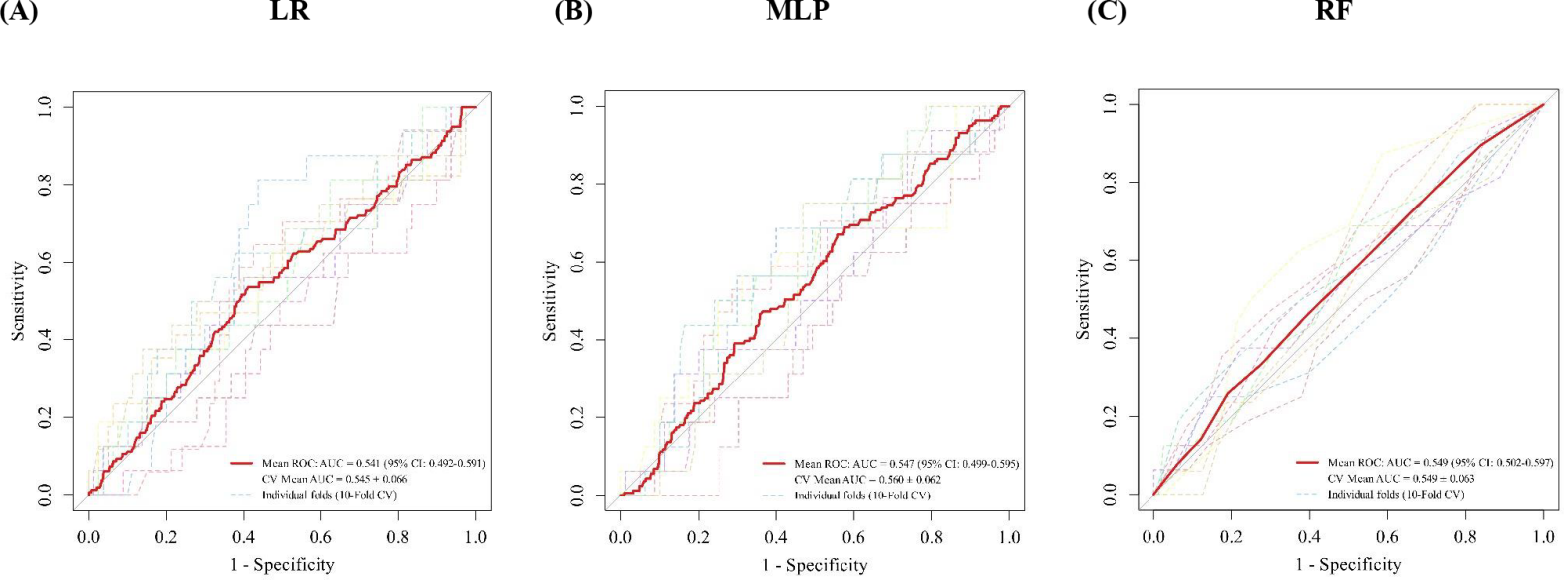
ROC curves of 3 models based on 10-fold cross-validation. **(A)** LR; **(B)** MLP; **(C)** RF. The red line represents the mean ROC curve, and dashed lines represent individual folds. The shaded area indicates the 95% confidence interval of the mean AUC.

The predictive performance of the three models was evaluated using 10-fold cross-validation (**Figure 1**). The mean AUC for LR was 0.541 (95% CI: 0.492–0.591), for MLP was 0.547 (95% CI: 0.499–0.595), and for RF was 0.549 (95% CI: 0.502–0.597). The cross-validation mean AUC values were 0.545 ± 0.066 for LR, 0.560 ± 0.062 for MLP, and 0.549 ± 0.063 for RF.

Although the overall discriminative ability was modest across all models, the MLP demonstrated slightly higher mean AUC and lower variability compared to LR and RF, suggesting relatively better stability across folds. The overlapping confidence intervals indicate that the differences among models were small.

**Figure 2:**
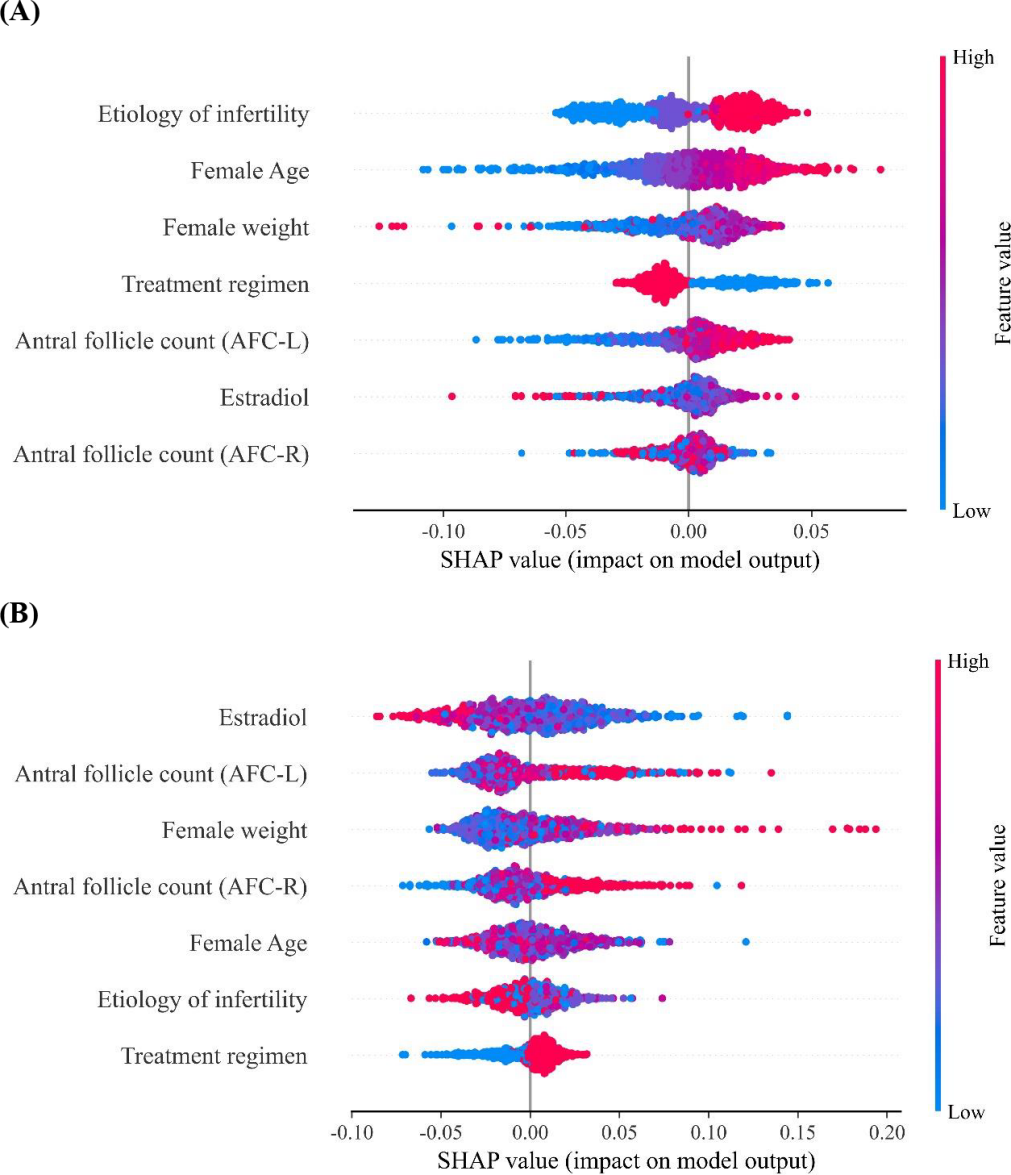
SHAP summary plots for feature importance in the MLP and RF models. **(A)** MLP model; **(B)** RF model. Each dot represents one observation. The color indicates the feature value (red: high; blue: low), and the horizontal axis represents the SHAP value reflecting the impact of each feature on the model output.

As shown in **Figure 2**, SHAP analysis was performed to assess feature contributions in the MLP and RF models. In the MLP model, etiology of infertility and female age were identified as the most influential predictors, followed by female weight and treatment regimen. In contrast, the RF model ranked estradiol as the most important feature, followed by AFC, female weight and female age.

Both models consistently identified female age and antral follicle count as important contributors, supporting the clinical relevance of these variables. The MLP exhibited more evenly distributed SHAP values across predictors, whereas the RF model showed greater dispersion, suggesting higher variability in feature impact across individual predictions.

**Figure 3:**
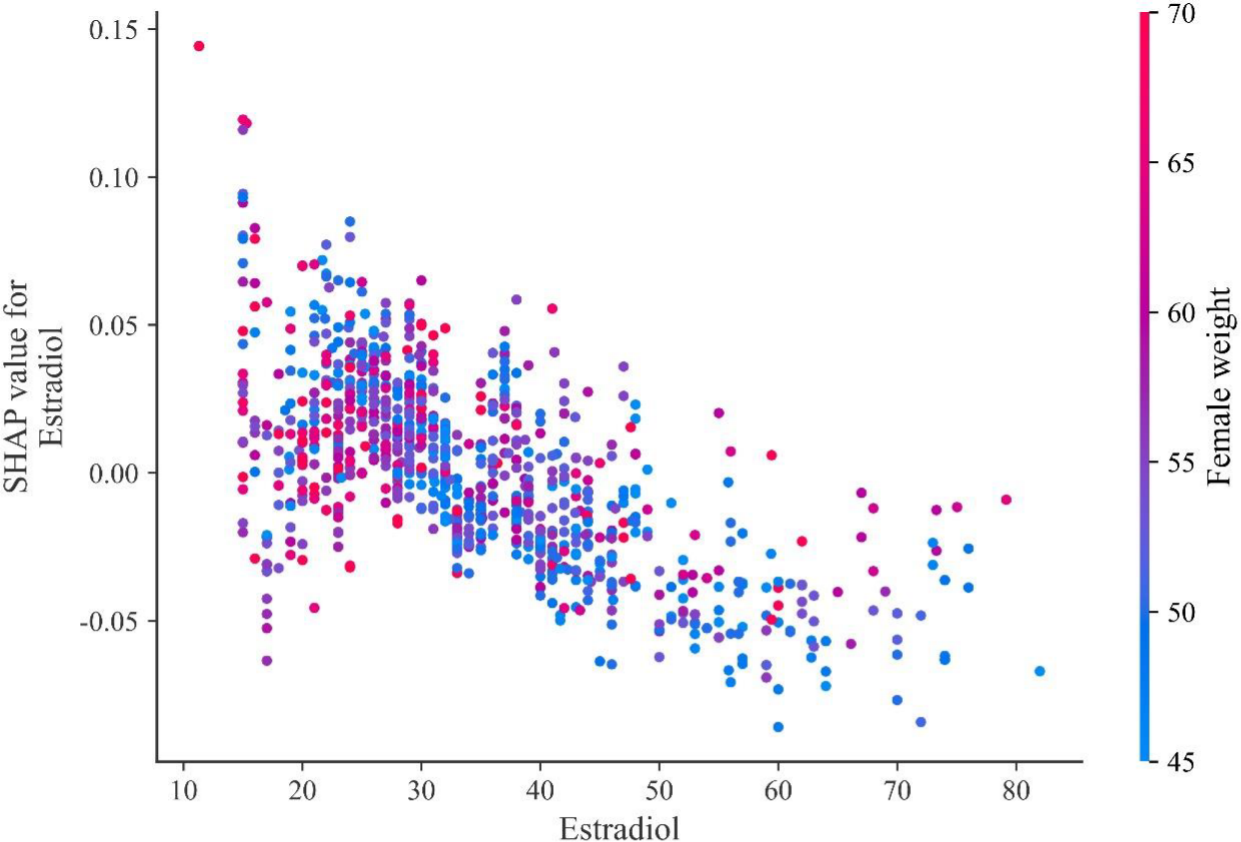
SHAP dependence plot for estradiol (E2) in the RF model.

The SHAP dependence plot for estradiol (E2) demonstrates a nonlinear association with model output (**Figure 3**). At lower E2 levels (approximately <40 pg/mL), SHAP values were predominantly positive, indicating a positive contribution to predicted pregnancy probability. As E2 levels increased beyond this range, SHAP values gradually decreased and became negative, suggesting reduced contribution at higher concentrations. The color gradient indicates female body weight. At similar E2 levels, individuals with higher body weight tended to show relatively higher SHAP values, suggesting a potential interaction between E2 and body weight in the model.

**Table 4** summarizes the performance of the LR, RF, and MLP models across 10-fold cross-validation. Overall, all three models showed modest and comparable discriminative ability. The AUC ranged from 0.562 to 0.633 for LR, 0.515 to 0.783 for RF, and 0.562 to 0.683 for MLP. Sensitivity and specificity varied across folds in all models, with RF showing greater variability. In comparison, the MLP demonstrated relatively more consistent AUC and corrected accuracy values across folds, suggesting improved stability.

**Table 4:** Performance of LR, RF, and MLP models across 10-fold cross-validation.

K10 Cross-Validation		1	2	3	4	5	6	7	8	9	10
LR	Threshold	0.42	0.42	0.4	0.32	0.46	0.38	0.42	0.32	0.48	0.42
	TP	12	11	10	11	12	12	10	12	10	11
	FP	41	40	42	46	33	54	48	60	30	41
	TN	39	40	38	34	47	26	31	19	49	38
	FN	5	6	6	5	4	4	6	4	6	5
	Sensitivity	0.706	0.647	0.625	0.688	0.750	0.750	0.625	0.750	0.625	0.688
	Specificity	0.488	0.500	0.475	0.425	0.588	0.325	0.392	0.241	0.620	0.481
	Accuracy	0.526	0.526	0.500	0.469	0.615	0.396	0.432	0.326	0.621	0.516
	Corrected Accuracy	0.597	0.574	0.550	0.556	0.669	0.538	0.509	0.495	0.623	0.584
	Youden Index	0.193	0.147	0.100	0.113	0.338	0.075	0.017	-0.009	0.245	0.169
	AUC	0.596	0.562	0.581	0.586	0.615	0.625	0.625	0.592	0.633	0.579
RF	Threshold	0.22	0.24	0.24	0.22	0.14	0.34	0.3	0.22	0.22	0.32
	TP	14	12	12	11	10	9	11	9	11	11
	FP	49	45	40	42	57	40	49	52	51	40
	TN	31	35	40	38	23	40	30	27	28	39
	FN	3	5	5	5	6	7	5	7	5	5
	Sensitivity	0.824	0.706	0.706	0.688	0.625	0.563	0.688	0.563	0.688	0.688
	Specificity	0.388	0.438	0.500	0.475	0.288	0.500	0.380	0.342	0.354	0.494
	Accuracy	0.464	0.485	0.536	0.510	0.344	0.510	0.432	0.379	0.411	0.526
	Corrected Accuracy	0.606	0.572	0.603	0.581	0.456	0.531	0.534	0.452	0.521	0.591
	Youden Index	0.211	0.143	0.206	0.163	-0.088	0.063	0.067	-0.096	0.042	0.181
	AUC	0.668	0.593	0.605	0.587	0.580	0.732	0.585	0.783	0.515	0.582
MLP	Threshold	0.36	0.34	0.38	0.26	0.48	0.24	0.32	0.2	0.42	0.32
	TP	12	12	11	11	11	11	11	14	13	12
	FP	41	42	40	42	36	52	42	63	49	39
	TN	39	38	40	38	44	28	37	16	30	40
	FN	5	4	5	5	5	5	5	2	3	4
	Sensitivity	0.706	0.750	0.688	0.688	0.688	0.688	0.688	0.875	0.813	0.750
	Specificity	0.488	0.475	0.500	0.475	0.550	0.350	0.468	0.203	0.380	0.506
	Accuracy	0.526	0.521	0.531	0.510	0.573	0.406	0.505	0.316	0.453	0.547
	Corrected Accuracy	0.597	0.613	0.594	0.581	0.619	0.519	0.578	0.539	0.596	0.628
	Youden Index	0.193	0.225	0.188	0.163	0.238	0.038	0.156	0.078	0.192	0.256
	AUC	0.596	0.598	0.562	0.581	0.590	0.594	0.581	0.683	0.645	0.601

MLP, Multilayer perceptron; RF, Random forest; LR, Logistic regression.

## 4 Discussion

In this study, LR, RF, and MLP models showed modest and comparable discrimination for predicting clinical pregnancy after IUI. In 10-fold cross-validation, the AUCs of the three models were close, with overlapping confidence intervals, suggesting that performance differences were limited. Therefore, the main potential utility of these models lies in exploratory risk stratification rather than standalone clinical decision-making.

This study found that at the default threshold of 0.5, the model prioritizes high specificity, resulting in reduced sensitivity and difficulty in identifying all potential pregnancies. However, adjusting the decision threshold improves sensitivity at the expense of specificity. In clinical settings, high specificity may be prioritized. Conversely, in high-risk populations requiring identification of all potential pregnancies, enhancing sensitivity should be the primary goal. These findings underscore the importance of adjusting decision thresholds based on clinical priorities and patient characteristics.

Infertility affects approximately 8–12% of couples worldwide, imposing considerable psychological and financial stress (7). In this context, data-driven models may serve as effective decision-support tools that complement, rather than replace, clinical judgment and reduce unnecessary transition to *in vitro* fertilization. Previous studies applying ML to IUI prediction have been limited. Wu et al. (8) developed a random forest–based model with 11 predictors and an accuracy of 60.8%, while Pavlovic et al. (9) reported 71.1% accuracy using a first-generation LVQ-ANN model. Direct comparisons across studies should be interpreted cautiously due to differences in patient populations, outcome definitions, and class imbalance. In our cohort, model performance was modest, and the main contribution of this study is a standardized comparison of LR, RF, and MLP under the same evaluation framework.

Among the key predictors, basal estradiol (E_2_) emerged as the strongest contributor. The role of E_2_ in predicting fertility outcomes remains controversial. Buyalos et al. (10) reported that elevated basal E_2_ (>80 pg/mL) was associated with reduced live birth rates, especially among women older than 38 years. In contrast, other studies, including those by Merviel et al. (11) and Mullin et al. (12), found no significant association between basal E_2_ and IUI pregnancy rates. Such discrepancies may stem from differences in study populations, ovarian response, and stimulation protocols. Notably, evidence from natural-cycle IUI further supports a potential prognostic value of early-follicular estradiol. In a study by Fukuda et al. (13), analysis of 163 natural-cycle treatment cycles showed that conception cycles had significantly higher day-3 estradiol levels than non-conception cycles (38 ± 26 vs 23 ± 18 pg/mL). Moreover, estradiol/androstenedione, estradiol/testosterone, and estradiol/FSH ratios were all markedly higher in conception cycles (29 vs 17, 2.3 vs 1.2, and 6.2 vs 3.6, respectively), with statistically significant differences for all indicators. These findings suggest that elevated estradiol and higher estradiol/androgen and estradiol/FSH ratios on cycle day 3 are associated with improved conception probability in subsequent IUI cycles. In our study, the strong contribution of E_2_ likely reflects its role as an indicator of ovarian reserve and early follicular activity, thereby influencing the overall likelihood of conception.

Female age was identified as the second most influential variable, consistent with numerous studies showing a negative correlation between maternal age and IUI success (14, 15). Age influences oocyte quality, endometrial receptivity, and sperm-egg interactions, while advanced paternal age may increase sperm DNA fragmentation and miscarriage risk. Similarly, AFC is a well-established marker of ovarian reserve and stimulation potential (16, 17). Higher AFC is generally associated with greater follicular recruitment and improved pregnancy outcomes, whereas reduced AFC reflects diminished ovarian reserve and lower reproductive potential. Body weight also emerged as an important predictor. Although several studies have reported no significant relationship between BMI and IUI outcomes (18), others demonstrated that obesity impairs ovulation, reduces oocyte quality, and increases miscarriage risk, especially in women with polycystic ovary syndrome (PCOS) (19). These findings highlight the need to consider both female and male BMI in clinical evaluation, as excessive adiposity may also affect semen parameters and treatment response (20). Encouraging weight optimization before IUI could therefore improve overall reproductive outcomes. The treatment regimen was also an important determinant of IUI outcomes. Although our study did not compare specific ovulation-induction medications, prior evidence shows that choosing between a natural cycle and an induced cycle has clear clinical implications. Multiple studies have reported higher pregnancy rates in stimulated cycles, particularly among younger women. Yin et al. found that the clinical pregnancy rate was significantly higher in stimulated cycles (14.69%) than in natural cycles (8.45%), with a greater benefit in women aged ≤35 years (15.94% vs. 9.09%) (21). Carpinello et al. similarly observed increased pregnancy rates in stimulated cycles among women aged 38–40 years (14% vs. 10%), although the possibility of a chance finding could not be excluded (22).

Other studies highlight specific subgroups that may benefit from stimulation. Li et al. reported that natural-cycle IUI is safe, but ovulation induction combined with IUI is advantageous for women with ovulatory disorders. They also noted that combining oral agents with gonadotropins can enhance pregnancy outcomes, with letrozole showing consistent effectiveness within these regimens (23). In patients with unexplained infertility, Wang et al. found that letrozole combined with low-dose HMG before IUI resulted in higher pregnancy rates and lower rates of multiple pregnancy and miscarriage, supporting this protocol as a safe and effective option (24).

However, findings in older women are more variable. Frank et al. showed that for women aged 38–43 years with diminished ovarian reserve, controlled ovarian hyperstimulation combined with IUI remained a feasible approach, yielding a clinical pregnancy rate of 7.5%. In this population, oral stimulation agents—particularly letrozole—were associated with the highest pregnancy rates (9.1%) (25). These results suggest that while stimulation tends to improve pregnancy outcomes in many patients, the magnitude of benefit may decline with increasing age or reduced ovarian reserve.

The MLP model was selected because IUI outcomes are influenced by multiple interrelated hormonal, ovarian, and demographic factors, for which nonlinear interactions are biologically plausible but difficult to capture using traditional linear models. As a classical neural network architecture, MLP provides a practical balance between modeling flexibility and clinical interpretability. The aim of this study was not to exhaustively compare all machine learning algorithms, but to evaluate whether a neural network based approach offers incremental value over commonly used clinical benchmark models, including logistic regression and random forest. More complex algorithms were not explored because of the limited sample size and the increased risk of overfitting, which may compromise generalizability. Within this framework, the MLP model demonstrated modest but consistent performance advantages, suggesting that accounting for nonlinear relationships may be beneficial for IUI outcome prediction.

In conclusion, the MLP model showed promising performance for predicting IUI outcomes, particularly in terms of balanced accuracy and AUC. However, the model’s performance is limited by the homogeneity of the study population and the potential lack of generalizability across different clinical settings. External validation is necessary to assess the model’s robustness and ensure its applicability in diverse populations and healthcare environments. Future studies should focus on validating the model in multi-center cohorts and investigating its performance in real-world clinical practice.

## 5 Limitations

This study has several limitations. First, although the dataset included 957 IUI cycles, the number of positive pregnancy outcomes was limited, resulting in class imbalance. While SMOTE was applied during training and a sensitivity analysis without SMOTE was performed, the impact of imbalance on model stability cannot be fully excluded. Second, the overall discriminative performance of the models was modest, suggesting that clinical pregnancy after IUI is influenced by factors beyond routine clinical variables. Third, although SHAP analysis improved interpretability for the MLP and RF models, detailed misclassification analysis was not performed. Finally, this was a single-center retrospective study, and external validation in larger, multi-center populations is required before clinical application.

## 6 Conclusion

This exploratory study developed an artificial neural network–based model to assess the likelihood of clinical pregnancy after IUI. The MLP showed modest and relatively stable performance compared with conventional models and identified several clinically relevant predictors, including basal estradiol, female age, infertility cause, body weight, and antral follicle count. Given the limited sample size and discriminative performance, these findings suggest that machine learning approaches may offer exploratory value for risk stratification and individualized assessment, rather than serving as a standalone tool for clinical decision-making in assisted reproduction.

## Data Availability

The raw data supporting the conclusions of this article will be made available by the authors, without undue reservation.
