# Supplementary material for: Development and evaluation of clinical pregnancy prediction models for intrauterine insemination using three machine learning algorithms
Source: Front Endocrinol (Lausanne). 2026 Apr 24;17:1749988. doi: 10.3389/fendo.2026.1749988 (PMC13152776; doi:10.3389/fendo.2026.1749988)
Supplement: Supplementary file 1 [file DataSheet1.pdf]

Supplementary Materials:

Development and Evaluation of Clinical Pregnancy Prediction Models for Intrauterine Insemination Using Three Machine Learning Algorithms

Table S1. Confusion matrices of the MLP, RF, and LR models in the IUI test set without SMOTE-based class balancing

Algorithm	Actual outcome	Predicted non-pregnant	Predicted clinical pregnancy
**MLP**	Non-pregnant (n=160)	160 (82.9)	0
	Clinical pregnancy (n=33)	33 (17.1)	0
	Total (n=193)	193 (100.0)	0
**RF**	Non-pregnant (n=160)	143 (74.09)	17 (8.81)
	Clinical pregnancy (n=33)	29 (15.03)	4 (2.07)
	Total (n=193)	172 (89.12)	21 (10.88)
**LR**	Non-pregnant (n=160)	158 (81.86)	2 (1.04)
	Clinical pregnancy (n=33)	32 (16.58)	1 (0.52)
	Total (n=193)	190 (98.45)	3 (1.55)

Footnotes: MLP = Multilayer perceptron; RF = Random forest; LR = Logistic regression.

Table S2. Classification performance of the MLP, RF, and LR models in the IUI test set without SMOTE-based class balancing

Algorithm	Accuracy (%)	Balanced accuracy (%)	Sensitivity (%)	Specificity (%)
**MLP**	82.9	50	0	100
**RF**	76.16	51.58	13.79	89.37
**LR**	82.38	50.89	3.03	98.75

Footnotes: MLP = Multilayer perceptron; RF = Random forest; LR = Logistic regression.
